# Charge accumulation in electron cryomicroscopy

**DOI:** 10.1016/j.ultramic.2018.01.009

**Published:** 2018-04

**Authors:** Christopher J. Russo, Richard Henderson

**Affiliations:** MRC Laboratory of Molecular Biology, Francis Crick Avenue, Cambridge CB2 0QH UK

**Keywords:** Low-dose electron microscopy, Single-particle reconstruction, Charging, CryoEM, Structure determination

## Abstract

When irradiated in a transmission electron microscope, plunge-frozen, amorphous water ice specimens accumulate a pattern of static charge that changes dynamically as the specimen is irradiated, and which can deflect the transmitted electrons and blur the resultant micrographs. Here we provide a physical description of this charge accumulation and characterise its dynamic behaviour in the context of low-dose electron cryomicroscopy (cryoEM). We observe the accumulation of positive charge in the primary irradiation area as expected from earlier work. To our surprise, we also observed a build-up of negative charge in nearby unirradiated regions of the specimen. Using a standard carbon support foil containing a pure water ice specimen, we collect a portion of this negative charge in the micrometer sized specimen holes which act as electrostatic lenses. These unusual, diverging micro-lenses are extremely sensitive charge detectors that allow us to directly measure the magnitude and dynamics of charge accumulation and neutralisation that occur during cryoEM imaging. Using these measurements, we find that the build-up of charge on the specimen saturates to a dynamic equilibrium at an electron fluence which is orders of magnitude lower than required for a typical low-dose micrograph. The measurements here will guide the development of optimal imaging conditions for biological specimens and contribute to a complete theory of information loss in electron cryomicroscopy.

## Highlights

• A physical account of charge accumulation in low-dose cryoEM is presented.
• We describe electrostatic micro-lenses that are extremely sensitive charge detectors.
• These micro-lenses allow the direct measurement of charge accumulation.
• Charge build-up saturates within the first millisecond of a typical micrograph.

## 1 Introduction

When irradiated with electrons of sufficient energy to traverse its thickness, a biological molecule suffers rapid and irreversible damage that destroys its structure [1], [2]. This limits the amount of information available in an electron micrograph of a biological specimen and ultimately determines the resolution at which its undamaged structure can be determined. It is important to consider all forms of image blurring or contrast loss that might occur during the acquisition of a low-dose image of a biological specimen under cryogenic conditions. The thin foils of amorphous carbon used in cryoEM are poor conductors at liquid nitrogen temperatures (resistivity of $10^{-2}-10^{-1}$ Ωcm) [3] and the suspended ice is an insulator which is many orders of magnitude less conductive (10^9^ Ωcm for crystalline pure water ice at 190K, 10^8^ Ωcm for crystalline 0.1 M NaCl ice at 190K) [4], which is part of the motivation for quantifying the amount of charging that occurs during imaging. Here and in an accompanying paper [5], we investigate one source of image blurring in cryoEM – the build-up and fluctuations of electric charge in, and on, the specimen during imaging.

Much is known about charging effects in the transmission electron microscope [6], [7], [8], [9]. When irradiated with high energy electrons in a localised area, semiconducting and insulating materials undergo ionisation events by several inelastic scattering processes and lose energetic electrons to the vacuum [10]. These take the form of emitted secondary electrons, most having an energy of about 1−100 eV, and which leave behind regions of positive charge that create electric fields in and around the specimen which can deflect the electron beam [10]. Over time, the positive charge builds up in the irradiated layer and can then shift the phase of subsequent electron waves. Sometimes called the Berriman effect [11], [12], this build-up of semi-static charge in the specimen occurs when an insulating or semiconducting material, like an amorphous carbon foil, is irradiated in a small region (1−10 µm in diameter) with an electron beam and then subsequently imaged with a broad, low-intensity beam ($10^{-2}$ e${}^{-}$/Å^2^) and high defocus (∼−10 mm). A region corresponding to the intense beam then appears dark in the highly defocused image; under continuing low flux imaging, the dark contrast then slowly decays away until it virtually disappears. This distinguishes it from the beam-induced build-up of hydrocarbons or other contaminants on the surface of the foil [13], which is permanent after irradiation. High-defocus renders the image sensitive to small deflections in electron path, like those that occur when a specimen becomes charged [7]. Models have been proposed to account for how charge builds up on semi-insulating specimens at or above room temperature [14], [15], [16] and even cryoEM specimens on carbon films [17], but no direct measurements of the charging phenomenon on cryoEM specimens under typical irradiation conditions have been reported. Here we use these high defocus conditions in conjunction with standard low-dose imaging methods to investigate the build-up of charge on a standard cryoEM specimen, consisting of vitrified water spanning the micron sized holes in an amorphous carbon foil, during typical imaging at 300 keV. Using an interesting observation of a micro-lensing effect, we are now able to quantify this build-up of charge and characterise the time and length scales involved in the build-up of charge during low-dose imaging in cryoEM.

## 2 Materials and methods

### 2.1 Specimen preparation

Specimens were prepared by plunge freezing using the Dubochet method [18], [19] and a manual plunger of the Talmon design [20]. Briefly, 3 µL of deionised, filtered water (resistivity  > 18 MΩcm) was applied to a specimen support (a patterned amorphous carbon foil on a gold grid, Quantifoil Au 1.2/1.3 [21]) in a cold room at 4 °C. The grid was blotted manually with filter paper (Whatman No. 1) from one side for 10 s in a humid chamber and then was plunged immediately into liquid ethane held at a temperature of 93K using a precision cryostat [22]. The specimen supports were rendered hydrophilic by exposure to a residual air plasma (glow discharge) in an Edwards S105B for 30 s, just prior to use. The frozen specimens were transferred to small plastic grid storage boxes and kept in liquid nitrogen until they were imaged in the electron microscope.

### 2.2 Electron microscopy

**Fig. 1:**
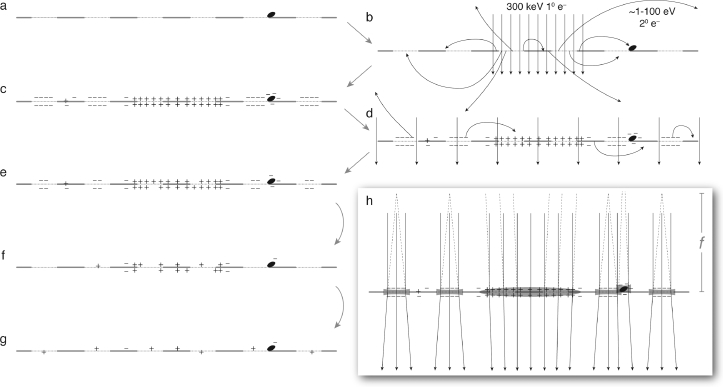
Diagram of charge build-up during electron irradiation of poorly conducting specimen. Left column (a,c,e,f,g) shows diagrams of the charge state of the specimen during different parts of the experiment, beginning with the neutral, uncharged specimen before irradiation (a). The grey line represents a section through the suspended carbon foil, which supports thin films of amorphous water ice (dotted lines) which for cryoEM specimen are typically of order 500 Å thick and  ∼ 1 µm in diameter. On the right are the two irradiation modes used in the experiment: a high-flux condition in a local region which is the same as typical low-dose imaging condition (b), and the very low-flux, broad beam used for search mode and here used with high defocus to detect the charge state of the specimen (d). The focused beam induces the generation of secondary electrons, many of which are redeposited on nearby regions of the foil and leave behind a region of positive charge. The broad beam condition in (d) is used to slowly neutralise the built up charge (e to f) until it returns to a state similar to the initial neutral configuration (g). Black dot is a surface contaminant particle that also accumulates charge during irradiation. Inset (h) shows a model of the micro-lensing caused by the charge build-up on the specimen, and corresponds to the state diagrammed in (c). Each suspended region of negatively charged ice creates a diverging, electron micro-lens with focal length *f*, which is used to measure the magnitude and sign of the charge build-up. Images in Figs. 2 and 3 are recorded in the focal plane that is at the top of (h). Note also that the central positively charged area causes convergence of the electron beam, resulting in an apparent increase in the diameter of the central region of the highly defocused micrographs in Figs. 2 and 3, as discussed in Section 3.2.

**Fig. 2:**
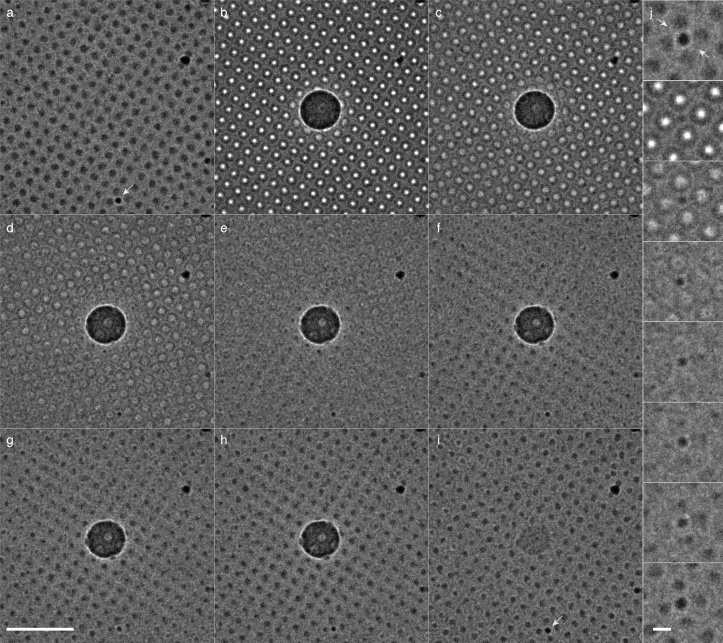
Charge build-up and neutralisation. Series of highly defocussed 300 keV images of a 30 µm square area of a holey carbon film with amorphous ice prepared by plunge freezing. (a) shows the area prior to illumination apart from the electron irradiation used to record this highly defocused, low magnification image (1.2 $\times 10^{-4}$ e${}^{-}$/Å^2^) and (b)-(i) shows successive images after irradiation of the centre of the square (5 µm diameter focused beam) by 1.4 e${}^{-}$/Å^2^. Panel b shows the image immediately after irradiation and frames (c)–(i) are after increasing amounts of illumination of the whole region, 1.2 $\times 10^{-4}$ e${}^{-}$/Å^2^ per image for b–h. The final frame (i) is the last frame in the series of 300, which is after 3.6 $\times 10^{-2}$ e${}^{-}$/Å^2^ of cumulative dose, corresponding to 25 min. The side panel (j) shows magnified views of a surface contaminant particle, which is shown by an arrow in (a) and (i). Scale bars are 10 µm and 1 µm; high intensity is white and low intensity is black in all panels.

Frozen specimens were transferred to the load lock system of an electron cryomicroscope (FEI Polara) and loaded into the specimen position. All micrographs were recorded using 300 kV bright field transmission mode, and detected with a 2048  ×  2048 phosphor-coupled CCD camera (Gatan Orius). Micrographs were collected using a pre-specimen beam blanker using the data collection software attached to the camera (Gatan Digital Micrograph), which allows capture of a series of micrographs using a set exposure time and a fixed delay between images. A diagram of the typical experiment is presented in Fig. 1. A low-dose experiment scheme was set up with two modes: *mode 1*: Low flux (1.2  ×  10${}^{-4}$ e${}^{-}$/Å^2^/*s*), low magnification (350 × ), broad, parallel beam (2000 µm^2^) and high defocus (−60 mm) *mode 2*: High flux (1.4 e${}^{-}$/Å^2^/*s*) focused beam (diameter = 5 µm) centred in the low flux region, exposed continuously for anywhere from 1 m sec to 100 s depending on the experiment. To conduct an experiment as diagrammed in Fig. 1, the specimen was moved to a new, unirradiated square on the support with the beam off, and then a single 0.2 s image in *mode 1* was collected, which becomes the first image in the movie. The microscope was then switched to *mode 2* and the centre region of the square was irradiated for the specified time corresponding to a particular fluence of electrons. The microscope was then switched back to *mode 1*, and a movie was collected by taking a series of 10–300 images (depending on the particular experiment) with a set delay between each image to allow the files to be written out. The delay for the supplementary movie and Fig. 2 was 5 s, and was limited by the bandwidth of writing the files to disk. Thus the total elapsed time was between 1 and 25 min depending on the experiment.

**Fig. 3:**
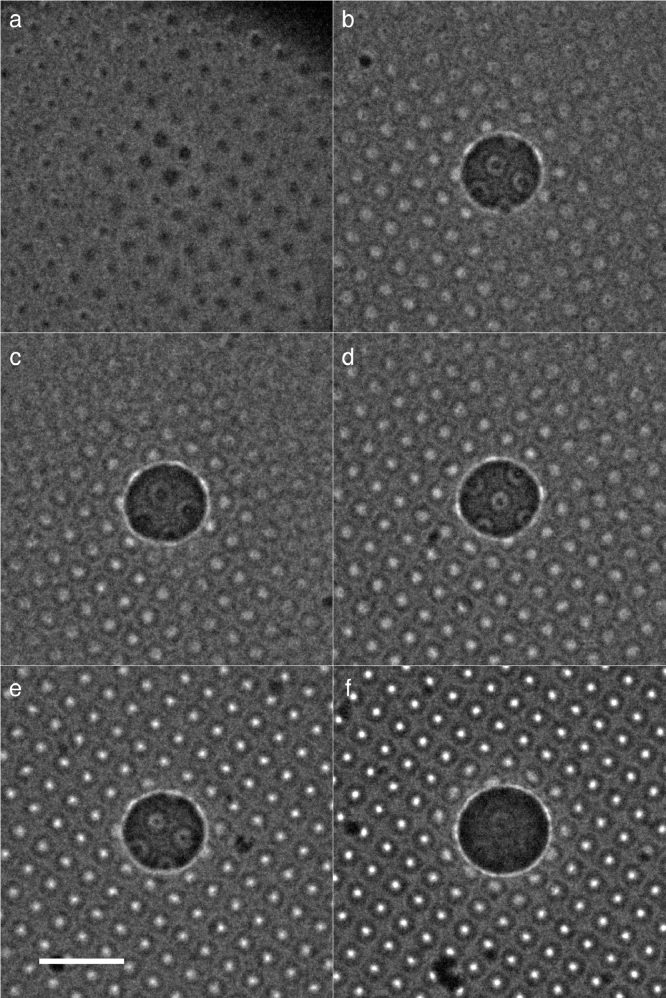
The build-up of charge during imaging. Representative images of Berriman build-up. Each panel shows a different region of the holey carbon with amorphous ice from different widely-separated grid squares immediately after irradiation with a  5 µm diameter primary beam using increasing electron doses. The image in each panel was recorded under the same conditions as used for Fig. 2b, with increasing doses of 4.7$\times 10^{-4},$ 1.4$\times 10^{-3},$ 1.4$\times 10^{-2},$ 1.4$\times 10^{-1},$ 1.4 and 14 e${}^{-}$/Å^2^ in the central region. The results of this series are analysed quantitatively in Fig. 4(c). Scale bar is 5 µm.

**Fig. 4:**
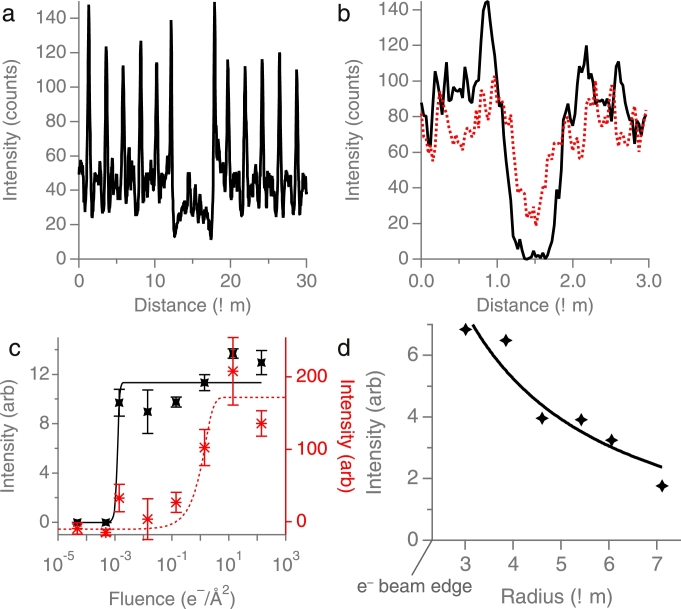
Quantifying the dynamics of charge build-up and neutralisation in cryoEM. (a) shows a line section along a row of holes from Fig. 2(b) with the primary irradiated area at 12–18 µm. (b) shows a line section across the image of the surface contaminant particle in Fig. 2j, with the solid black line from the first panel and the dotted red line from the second panel. (c) shows the build-up of positive charge (Berriman effect) in the primary area (black x) and the build-up of negative charge (inverse Berriman effect) in the adjacent unirradiated regions (red*), as a function of the primary fluence (see also Fig. 3). Note that the final point in the inverse Berriman effect (red*) is lower because the microlenses have charged up enough so that their focal point is now below the focal plane of the image. Plot (d) shows the approximate range of secondary electrons emitted from the primary area before saturation (corresponding to Fig. 3c). The line is a fit with 1/*r* dependence, to the data as described in the text, where *r* is the distance to the centre of the primary irradiation area.

This experimental scheme was tested on holey amorphous carbon foils having an additional carbon film on top of the foil, to establish the appropriate conditions, and then conducted on cryo-specimens prepared as described above. To determine the focal length of the microlenses we repeated the experiment several times under the same conditions until the maximum intensity for the focused microlenses was found; in this case - 60 mm. The error in determining this maximum is estimated to be  ± 5 mm. The beam current was measured using the current amplifiers attached to the two phosphor viewing screens, which had been previously calibrated against a picoammeter attached to a Faraday cup in the projection chamber. The total error in the measurement of the beam current, which is the dominant source of error in the reported flux, is estimated to be less than 30%. All other errors are less than 10%.

### 2.3 Data analysis and processing

Images were converted to MRC format and cropped, converted to 8 bit depth and saved as TIFF files for creating the panels in Figs. 2 to 3. In both figures, higher electron intensity is shown as white. Linear sections in intensity along lines were taken from the raw, unprocessed micrographs and used to create the plots in Fig. 4. To quantify the charge on a local region of the specimen, like the suspended ice within the holes of the foil, the normalised intensity vs. radius from the centre of the hole was found by integration about the azimuth, and the difference between the maximum at the centre of the hole and the minimum at the edge was taken as the intensity, as plotted in Fig. 4c. Although the thickness of the ice is not necessarily the same across the entire width of each hole, the image contrast due to charging is much greater than any variation due to ice thickness. This was repeated three times on each of three squares for three separate experiments, and the mean and standard deviation were taken as the representative data point and error bar, respectively. Supplemental movies of the charging process were created by combining the series of micrographs into a stack, which was drift corrected [23] and converted and compressed to .avi format using ImageJ [24].

## 3 Results

### 3.1 Imaging charge build-up and neutralisation

To investigate the kinetics of charge build-up and neutralisation, it was important to use specimens consisting of amorphous ice on a holey carbon film, which is the specimen used in most single particle cryoEM experiments [18]. We used electron-optical conditions for illumination and imaging that are equivalent to typical low-dose conditions used for single particle cryoEM, and which are similar to those used originally [11] to describe the Berriman effect. In particular, we used high defocus (−20 to −80 mm) at low magnification (350 × ) to amplify the small electrostatic deflections of the illumination that are caused by the charging. We found that −60±5 mm defocus maximised the intensity of the bright spots in the centre of the holes with amorphous ice. This can be used to estimate the focal length of the electrostatic lens and thus the lateral electric field at the hole.

### 3.2 Quantifying the charge

For a 300 keV electron to be displaced by 0.6 µm (*r*, the radius of the hole) in f=60 mm, this corresponds to a deflection angle of θ=arctanr/f=10 µrad. Using this information, an order of magnitude approximation for the electrical potential in the centre of the hole can be determined, in a way that follows previous estimates [6], [7]. A 300 keV electron has a velocity in the lab frame of $v_{e}=0.77c$ where *c* is the speed of light in vacuum. Thus the lateral velocity, *v_s_* required to give a deflection angle of 10 µrad is 2  ×  10^3^ m/s, which is imparted on the electron during its transit through the electric field of the charged specimen. If we take the vertical distance over which the lateral field is experienced by the electron as approximately 5 µm, which is approximately the distance between holes, then to accelerate the electron to this velocity over this distance, a field of 0.6  ×  10^6^ V/m is required. Thus the potential drop from the centre of the suspended ice to the edge of the hole in the foil is −0.4 V. We note that using a series of images at different defocus values might allow a more accurate analytical calculation of the potential [25], which would be desirable for a more detailed description of the electrostatic microlenses. A similar calculation for the central Berriman spot gives a potential of approximately +1 V after $10^{-3}$ e${}^{-}/$Å.

An explanation of how regions of positive or negative charge affect this highly defocussed image is given in Fig. 1h. The key panels illustrating the approach taken in all sections of this paper are in Fig. 2(a) and (b), which show images immediately before and immediately after illumination by the primary irradiating beam.

The region of positive charge in Fig. 2(b) shows up as a dark region because the positive electrostatic charge acts as a convergent lens. In contrast, the surrounding regions display bright spots on each hole containing amorphous ice, which represent divergent electrostatic lenses due to build-up of negative charge on these insulating regions. This negative charge is created by the deposition of low-energy secondary electrons that are emitted from the primary irradiated area. Sections through the micrographs are shown in Fig. 4a–b. The remaining panels show how both the Berriman (central positive patch) and inverse Berriman (surrounding negative charge accumulation) effects fade with successive low flux illumination. The final panel (Fig. 2i) shows an image that is very similar to the first image (Fig. 2a), with slightly darker intensity in the holes, likely due to the ice accumulating slightly more positive charge than the foil. The build-up of both the positive and negative charge on the specimen is quantified in Fig. 4c, where the fluence in *mode 1* (see Section 2.2) was varied over 7 orders of magnitude, from 10${}^{-5}$ to 10^2^ e${}^{-}$/Å^2^. Additional evidence in support of the microlensing observation is shown in Supplementary Fig. 1, which shows a similar montage to Fig. 2b–e, on a grid square that also contains some empty holes. It is also interesting to note in both Figs. 2 and 3 that there are even weak lensing effects within the primary irradiation area of the Berriman footprint, and we note that the diameter of the central Berriman patch gets slightly smaller as the charge fades in Fig. 2 and slightly larger as the charge increases in Fig. 3, indicating a change in the electrostatic lensing by the central region, but further work is required to quantify these.

### 3.3 Secondary electron range

To determine the range of secondary electrons landing on the specimen after being emitted from the primary region, we selected a primary fluence that did not charge up the entire square to saturation, but showed a distribution of charge intensity across the foil (Fig. 3c). We then quantified the amount of charge by integrating the intensity of the spots as a function of radius from the centre, and the result is shown in Fig. 4d. A curve was fit to the values where the intensity, and thus the magnitude of accumulated charge, falls off roughly as 1/*r*, as one might expect from geometric considerations alone. A fit to $r^{-2}$ gives a worse fit, but Fig. 4d provides only a rough estimate of the distribution. We find that the amount of accumulated negative charge falls to half its initial value in 3 µm. This is consistent with previous estimates made by repeated adjacent irradiation of the same specimens. The fact that all of the lenses in a square eventually charge up to a potential indicates that some small proportion of the emitted electrons travel long distances ( > 10 µm) across the specimen before they are deposited back on the surface and can easily be detected by this method.

### 3.4 Charge build-up on surface particles

Fig. 2j shows an enlarged view of a contaminant particle on the surface of the specimen support and demonstrates another interesting charging phenomenon. In the initial image, the particle appears dark and has a diameter of 1.0 µm (Fig. 4b, black curve). When the central region is irradiated, thus showering the surface of the specimen with secondary electrons, the particle now appears to be half as large (0.5 µm, dotted red curve). As the specimen is discharged through continued irradiation with the broad beam, the particle returns to its original size in the image. This is explained, much the same as the lensing effect within the holes, by charging of the particle. The negative charge attaches to the non-conductive particle and thus acts as a lens on the beam. As electrons transit the specimen, those near the particle are deflected radially away, thus mimicking the action of a small diverging lens (Fig. 1h). This phenomenon likely occurs on any contaminant particles that are not both conductive and in good electrical contact with the specimen support grounding electrode.

## 4 Discussion

The goal of the experiments reported here was to estimate quantitatively the magnitude and range of charging effects on typical ice-embedded specimens used for single particle cryoEM. Since beam-induced image blurring is a serious problem in such cryoEM images, it is important to estimate the relative contributions of specimen charging in comparison to mechanical motion induced by radiation damage. Both problems occur with typical ice-embedded biological structures because the ice matrix is an insulator and both the ice and the biological macromolecules undergo radiation damage consisting of the breakage of chemical bonds followed by diffusion and evaporation of volatile fragments.

As a result of the experiments reported here, we can describe the processes of charge build-up and neutralisation quantitatively. In an accompanying paper [5], we further describe the effect of microscopic charge fluctuations on the contrast in high resolution images, and these results contribute towards the development of a broad understanding (“the Grand Scheme”), describing the relative contributions of all detrimental phenomena that are currently limiting the scope of high resolution single particle cryoEM. We find that the primary electron irradiation that is typical of a low-dose exposure creates a region of positive charge (the Berriman effect) by loss of secondary electrons, with a final potential of approximately 1 V. We note that this potential is well below the work function of the ice ( ∼ 5 V) and would not prevent the emission of secondary electrons whose energy can be up to  ∼ 20–50 eV. Still, the potential may reduce the total number of secondary electrons emitted since their most probably energy is only 1–2 eV. The results shown in Fig. 4(c) show that most of this charge builds up during the first  ∼ 0.001 e${}^{-}$/Å^2^ with a smaller additional 10–20% increase as the fluence increases to  ∼ 1 e${}^{-}$/Å^2^. In contrast the build-up of negative charge on areas surrounding the irradiated area takes longer to reach a similar magnitude, presumably because the range of the secondary electrons is much larger in area than the primary irradiated area, the emitted secondary electrons do not all end up on the specimen, and not all the scattered electrons escape the irradiated region. Furthermore, the low energy secondary electrons will accumulate on the surface of the specimen, whereas the positive charge in the Berriman footprint will be distributed throughout the specimen thickness. The observation of the build-up of negative charge on surrounding areas is not surprising since the secondary electrons emitted from the primary irradiated area must land somewhere and the surrounding area is the nearest surface. The estimate of charge build-up of about +1 V in the central region and about −0.4 V on the surrounding regions, is similar to the charge estimated at 1.6 V by Reimer [26] for charge build-up on 2–3 µm sodium chloride crystals, which are in less intimate contact with the carbon film.

The implications for normal cryoEM imaging of the build-up of positive charge on the illuminated area during a typical cryoEM exposure together with the accompanying negative charge on the surrounding region mean that there may be some information lost during the first fraction of an electron per square Ångstrom of a typical exposure prior to the point at which the overall charge on the specimen reaches equilibrium. Accompanying the charge build up, we would expect a perturbation of the electron wave-front that would translate into a change in the defocus and therefore in contrast transfer function. This might partly explain the defocus offset that has been noticed when images of ice-embedded specimens in holes in carbon foils are compared with those recorded from similar ice-embedded specimens in holes in gold foils. The precise degree of defocus under different conditions should be measured accurately to test this but this is beyond the scope here.

The build up of positive and negative charges on these ice-embedded, non-conducting specimens which we have observed so clearly using 300 keV illumination would be expected to create even stronger perturbations of electron images using lower energy illumination. In particular, it is likely that any contrast observed in images of non-conducting, organic or biological specimens recorded using very low energy ( ∼ 100 eV electron illumination), is likely to be dominated by the charge that builds up on and around the illuminated area. Thus the contrast observed by Longchamp et al. in recent low-energy holography experiments [27] is likely to be due to surface charge accumulated on the vacuum dried, non-conducting structures. This is much like the charge that builds up on the particle in Fig 3j when it is irradiated with secondary electrons having an energy of  ∼ 10 to 100 eV.

Another interesting question to consider in this context is whether the influence on the quality of TEM images of electrostatic charge build-up on the specimen would be expected to be less if a microscope with a more powerful objective lens was used. In typical electron cryomicroscopes, the focal length of the objective lens is a few millimetres, with $C_{s}=C_{c}=2$ to 3 mm. Since the focussing of electrons by stronger magnetic lenses, such as the FEI ultratwin with a power about 3  ×  higher and $C_{s}=C_{c}=0.7$ mm, is carried out over a shorter physical distance between object and image, it would be expected that the effect of electrostatic lensing due to charge accumulation would be less if such a more powerful magnetic objective lens was used. We consider this question again in the context of dynamic charge fluctuations in the accompanying paper but, this will require future experimentation to quantify accurately.

Spotscan imaging has been advocated [28], [29], [30], and shown [31], [32] to bring a significant advantage in electron crystallography especially when recording images from tilted 2D crystals to obtain a 3D structure. Still, electron crystallographic imaging of 2D crystals is always carried out with the specimens on continuous carbon foils meaning these results are not directly mappable to single particle imaging methods. Specifically, the use of spotscan imaging on ice-embedded specimens in holey carbon or gold foils results in much greater amounts of electrostatic charge build-up than that reported in this paper because the illuminating beam has a smaller diameter than the holes in the holey foils so for at least part of the exposure the spot illuminates an area with no conductive path to the surrounding metal of the grid. Berriman and Rosenthal [33] have shown that a paraxial charge compensator can provide a source of secondary electrons for such specimens, and that this can at least partly neutralise the region of primary positive charge that otherwise builds up on the illuminated spot region. The mechanism of this neutralisation is thought to be via secondary electrons that traverse through the vacuum and land on the positively charged region, in much the same way as observed here for the inverse Berriman effect. It would be interesting to pursue this type of paraxial charge compensation quantitatively, but the results reported here suggest it might be possible to use a spot scan–like imaging geometry to minimise beam-induced mechanical motions without producing a deleterious degree of specimen charging. Still, the rate of secondary electron capture by the central region must match the generation of positive charge precisely, so this should be considered in designing such an illumination geometry. Our observations of the range of electron generation also support the idea that symmetrically illuminating the supporting foil around the specimen [3], particularly if it is made of a metal with high secondary yield like gold, may also help neutralise the positive charge that builds up since a high proportion of electrons emitted from the foil will be captured on the central insulating region being imaged.

Finally, the electrostatic micro-lensing phenomenon that has allowed us to estimate the charge build up due to the inverse Berriman effect may turn out to be useful for other types of investigation on different substrates or devices, such as those fabricated with common insulating materials like SiO_2_ or Si_3_N_4_. We have shown that a small disk of insulating material, when appropriately charged, can act as both a converging and diverging electron microlens. We envision that structures such as these could be incorporated in other devices to locally detect secondary electrons in specific regions and even create electron micro-optical devices.

## 5 Conclusions

From these measurements we conclude that charge, in the form of low energy secondary electrons, can accumulate on insulating regions of the specimen in the electron microscope even tens of microns away from the primary beam. This charge can be measured by incorporating insulating structures within the specimen that act as microlenses for a particular region of a low flux, parallel beam. Further, the positive charge left behind after the loss of secondary electrons saturates on a typical cryoEM specimen after between $10^{-3}$ and $10^{-1}$ e${}^{-}$/Å^2^. This means that the positive charge that builds up in the irradiated region of a cryoEM specimen saturates at a fluence that is much less than that incurred even in the first frame (typically with a dose of  ∼  0.1 e${}^{-}$/Å^2^) of a low-dose micrograph on a direct electron detector. Slight increases in potential can occur after this but are thought to be small and mostly related to the charge-up of regions within the low intensity tails of the beam. This positive charge is a likely physical explanation for offsets in defocus that are often observed in micrographs of ice imbedded specimens but further work is required to quantify this accurately and compare it to the physical movement of the ice layer. It is still not possible to say unequivocally that specimen charging under these conditions has no effect on image quality; it is possible that there will be a small defocus change during the first $10^{-1}$ e${}^{-}/$Å^2^ of the exposure, but based on the measurements here it is thought to be much less than the movement of the amorphous water at the onset of irradiation. While here we have considered the steady-state charge that accumulates on the specimen, it will be accompanied by a fluctuating charge that occurs due to the discrete nature of the electron scattering events and we consider this aspect of specimen charging in the accompanying paper [5].

## Appendix A Supplementary materials

Supplementary Data S1 Supplementary Raw Research Data. This is open data under the CC BY license http://creativecommons.org/licenses/by/4.0/. Supplementary Data S1

Supplementary Data S2 Supplementary Raw Research Data. This is open data under the CC BY license http://creativecommons.org/licenses/by/4.0/ Supplementary Data S2
