# Supplementary material for: Charge accumulation in electron cryomicroscopy
Source: Ultramicroscopy. 2018 Apr;187:43–9. doi: 10.1016/j.ultramic.2018.01.009 (PMC5862658; doi:10.1016/j.ultramic.2018.01.009)
Supplement: Supplementary Data S1 — Supplementary Raw Research Data. This is open data under the CC BY license http://creativecommons.org/licenses/by/4.0/. [file mmc1.pdf]

Supplementary Material

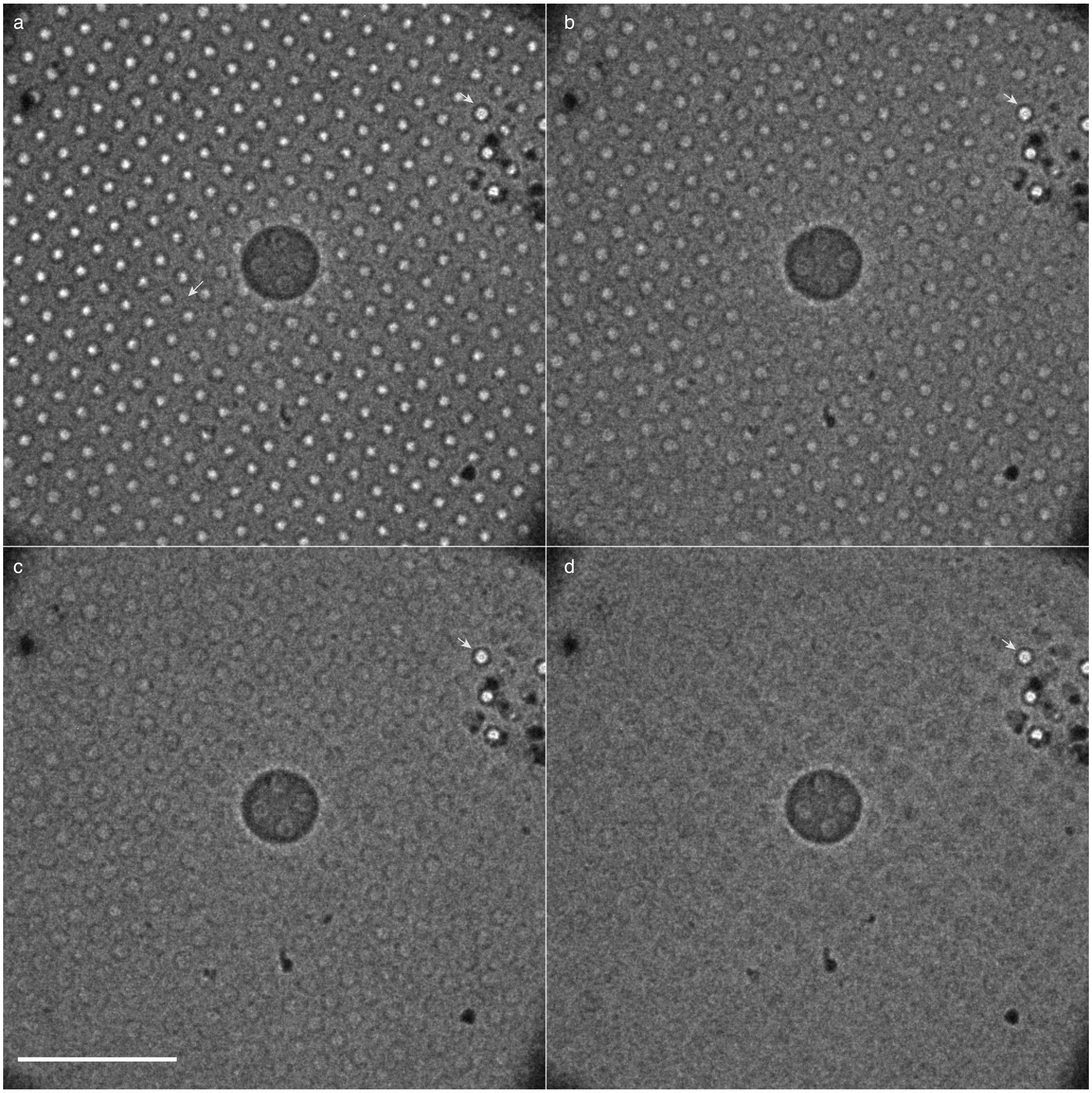

Supplementary Fig. 1: **Charge microlensing does not occur in holes without ice.** Panels (**a**)–(**d**) show the first four frames of a series, acquired under the same conditions as for Fig. 2, but where some of the holes in the foil are empty (arrow) and the defocus is -20 mm. Scale bar is 10 μm.

Supplementary Movie 1 The movie is the complete time series of images presented in Fig. 2, where the field of view is cropped to $(27\ \mu\text{m})^2$ and the frames have been aligned and compressed to create the $(512\ \text{px})^2 \times 8$ bit movie file.
